# Adverse Effects of Steroid Therapy in Sudden Sensorineural Hearing Loss: A Scoping Review

**DOI:** 10.1111/coa.14339

**Published:** 2025-05-30

**Authors:** M. Achanta, P. Kasetti, M. Fortune‐Ely, T. Ross, T. Magos, J. G. Manjaly

**Affiliations:** ^1^ Imperial College Healthcare NHS Foundation Trust London UK; ^2^ School of Medicine Imperial College London London UK; ^3^ University College London Hospitals NHS Foundation Trust London UK

**Keywords:** communication, informed consent, medico‐legal, pharmacology, sensorineural hearing loss

## Abstract

**Objective:**

Sudden sensorineural hearing loss (SSNHL) is an otologic emergency and is treated with steroid therapy. Despite adverse events (AEs) associated with long‐term steroid use being well evidenced, there is sparsity of literature regarding the AEs of short‐course prescriptions in the SSNHL cohort, which limits the quality of patient counselling and informed consent.

**Method:**

A literature search was performed on the Medline and Embase databases for studies assessing AEs in adults with SSNHL managed with oral (OST), intratympanic (ITS) and intravenous steroid therapy (IVS). Two authors screened titles, abstracts and full‐text articles, with conflicts resolved by a third reviewer. Forty‐three papers were included.

**Results:**

In systemic steroid therapies, hyperglycaemia and hypertension are reported in up to 29.8% and 37.9% of patients respectively. Patients with medication‐dependent diabetes and hypertension are at higher risk. Gastric and mood disturbances affected up to 27.9% and 44.6% of patients respectively. ITS carried risks of otalgia (up to 54.3%), dizziness (up to 27.1%), perforations (up to 11.5%) and otitis media (up to 4.7%).

**Conclusion:**

Comprehensive counselling is key in obtaining informed consent, especially in cohorts with diabetes mellitus (DM) and hypertension where monitoring of glucose and blood pressure is recommended. Gastroprotection should be considered. Future focus is required to study short‐term steroid AEs and raise awareness among prescribing clinicians and patients.

**Summary**

- Medication‐dependent diabetic and hypertensive patients are at increased risk of hyperglycaemia and hypertension with systemic steroids.
- Diabetic and hypertensive patients should be monitored while taking systemic steroids. GPs and community diabetic teams should be involved in titrating medications accordingly.
- ITS as first‐line SSNHL management in diabetic and hypertensive cohorts requires further investigation.
- PPI should be considered in patients at risk of gastrointestinal bleeding or dyspepsia.
- Information leaflets should be developed and supplied alongside urgent prescriptions.

## 1 Introduction

SSNHL is an otologic emergency typically characterised by sensorineural hearing loss over three consecutive audiometric frequencies developing over a period of up to 72 h [1]. Most cases (estimated to be 90%) do not have a known causal event, and given the broad demographic affected, are likely to be the manifestation of multiple aetiologies with similar clinical presentations. Theories include viral inflammation or ischaemic insult to the cochlea. The intention is to improve function through anti‐inflammatory and immune‐modulatory effects, thus reducing inflammation in the vestibulocochlear nerve and cochlea [2, 3]. SSNHL affects 5 to 20 people per 100 000, though under‐reporting, under‐investigation and delayed presentation mean that its epidemiology is difficult to ascertain [4].

Patients usually present to primary care or to the emergency department. As per ENT UK, first‐line treatment is OST of 1 mg/kg up to 60 mg for 7 days. This may then be tapered by 10 mg daily [1]. ITS is an alternative route of steroid administration, and several papers demonstrate equivocal benefit when compared to OST [5, 6]. The SeaSHeL national prospective cohort study recently examined SSNHL patients' demographics, management regimes, and clinical pathways, and revealed more understanding of risk factors such as cardiovascular disease and concurrent vertigo [7]. The STARFISH Trial, which started patient recruitment in January 2023, aims to compare hearing outcomes in SSNHL patients managed with OST versus ITS [8].

The side‐effect profile of long‐course steroids is well understood with reports of hyperglycaemia, hypertension, mood and sleep disturbance, thinning of skin and easy bruising [9]. However, the risks of shorter courses are poorly captured by clinicians and perhaps under‐reported by patients. Studies are often too heterogenous to the SSNHL patient base, both with respect to duration and intensity of steroids and their comorbidities, to draw valid comparisons. Xie et al. (2003) found an increased incidence of hypertension, diabetes and hyperlipidaemia in SSNHL patients. The incidence of hypertension ranged from 21.1% to 39.2% of SSNHL patients, and that the prevalence of SSNHL in diabetic patients is 1.54 times that of their nondiabetic counterparts [10].

With steroid usage being commonplace in ENT, a thorough understanding of their AEs is also necessary for general practitioners and emergency medicine clinicians. By synthesising the available data, we hope to provide clinicians with a better understanding of the associated risks, enabling them to make informed decisions and conduct more robust counselling when managing SSNHL. Our recommendations have been formed in collaboration with pharmacist colleagues.

### 1.1 Objectives of This Scoping Review

In performing this scoping review, we aim to establish the documented adverse effects of systemic and intra‐tympanic steroid therapy in the management of SSNHL.

### 1.2 Objectives

1. Detail the AEs of steroid therapy in SSNHL management.
2. Compare the AEs of systemic and ITS for SSNHL.
3. Provide recommendations for clinicians prescribing steroids for SSNHL.

## 2 Methods

### 2.1 Search Strategy

The Preferred Reporting Items for Systematic Reviews and Meta‐Analyses extension for Scoping Reviews (PRISMA‐ScR) guidance was followed. A literature search of the MedLine and Embase databases was performed in July 2024 to include articles up to July 2024. Subject headings were utilised to explore synonyms.

### 2.2 Inclusion Criteria

1. Papers exploring the management of SSNHL with OST, IVST, or ITS, and reported AE rates.
2. Adult patients (≥ 18 years)
3. Human studies.
4. English language.

### 2.3 Exclusion Criteria

1. Papers where AEs were not attributed to steroid therapy (i.e., AEs grouped with nonsteroid comparison arms).
2. Case reports.
3. Alternative middle‐ear steroid delivery (e.g., eustachian tube catheterisation).
4. Refractory SSNHL.
5. SSNHL management of patients with renal or hepatic impairment.

### 2.4 Selection of Sources of Evidence

Reports were exported to Covidence (Veritas Health Innovation Ltd.), a systematic review management software. Duplicate studies were manually and automatically identified and removed. Titles and abstracts were screened by two authors (M.A. and P.K.) and conflicts were resolved by a third author (T.R.).

Data relating to study design, steroid administration and AEs were extracted according to a template. Studies were grouped according to route of steroid administration. Steroids were converted using an online medical calculator (MDCalc Ltd.) to prednisolone; 10 mg of prednisolone was equivalent to 1.5 mg of dexamethasone, 40 mg of hydrocortisone or 8 mg of methylprednisolone. Where doses were given per kilogram and tapers were left undefined, the average weight was estimated to be 70 kg and tapers were estimated to allow for analysis. A formal meta‐analysis on the data was not possible due to heterogeneity.

## 3 Results

**FIGURE 1:**
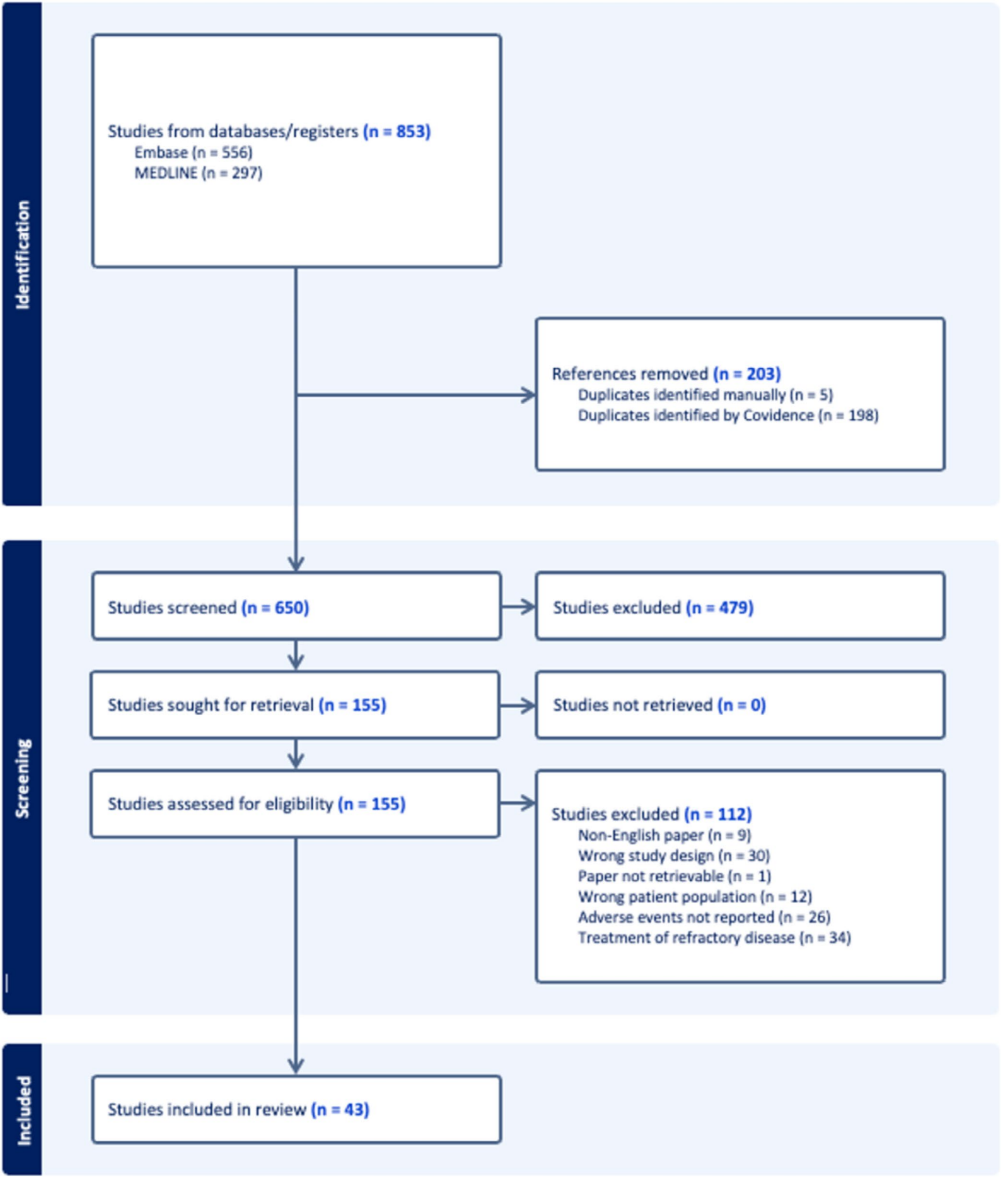
Preferred reporting items for systematic reviews and meta‐analyses flowchart illustrating how studies were screened.

Figure 1 illustrates the PRISMA flowchart. In total, 853 studies were identified, and 203 were duplicates. In all, 650 studies were screened, and 155 full texts were evaluated. Finally, 43 studies were included.

The most common design was randomised‐controlled trials (*n* = 17), followed by non‐ or quasi‐randomised trials (*n* = 10). A total of 32 papers collected prospective data. The median number of study participants was 68 (range: 6–908), and mean age was 50.6 ± 8.7. AE monitoring methodology was specified in 18 papers. In all, 16 papers reported participants' comorbidities.

We identified 22 ITS, 19 OST, 13 IVST, 7 combined OST and ITS, and 7 combined IVST and ITS study arms. Summary tables of patient demographics are included as supporting information (S1–S6) for conciseness.

### 3.1 Oral Steroid Therapy

In all, 18 reports were identified. (Table 3) 20 study arms are included, as one report compares high‐ and regular‐dose OST [11] and another compares steroid types [12].

Most studies (*n* = 11) used prednisolone. One paper did not report the steroid nor regimen [2]. The average daily dose of prednisolone was 63.0 mg (range 22.5–400.0 mg) and the average duration of therapy is 11.6 days (range 5–19 days). Seven papers specified dosages per weight, and three did not specify tapers.

Hyperglycaemia and hypertension are the most reported AEs with a range of 2.1%–29.8% and 4.2%–37.9% respectively. Severe hyperglycaemia occurred in one participant, who was removed from analysis [13]. Gastric disturbance and psychological disturbances, including altered mood and sleep, range from 4.2% to 27.9% and from 8.4% to 44.6% respectively. (Table 1).

**TABLE 1:** Adverse events in oral steroid‐only arms.

	Hyperglycaemia (%)	Hypertension (%)	Gastric disturbance (%)	Insomnia or mood disturbance (%)	Severe hyperglycaemia (%)
Koo 2016	1/24 (4.2)	1/24 (4.2)	1/24 (4.2)	Sleep: 2/24 (8.4)	
Han 2009	1/48 (2.1)				1/49 (2.0)
Rauch 2011	36/121 (29.8)		Appetite: 28/121 (23.1)	Mood: 54/121 (44.6)	
			Weight change: 22/121 (18.2)	Sleep: 44/121 (36.4)	
Halevy 2022	32/143 (22.4)	21/143 (14.7)			
Choi 2014 (normal dose)	17 (25.8)	17/66 (25.8)	14/66 (21.2)	Sleep: 5/66 (7.6)	
Choi 2014 (low dose)	6 (14.0)	15/43 (34.9)	12/43 (27.9)	Sleep: 1/43 (2.3)	
Plontke 2024 HD‐Dex	12/102 (11.8)	32/94 (32.0)			
Plontke 2024 Pred‐Control	3/99 (3.0)	36/95 (37.9)			
Kara 2010	6/31 (19.4)				

Halevy (2022) found there was a relative risk of 0.59 versus 0.13 (*p* < 0.001) of hyperglycaemia in DM versus non‐DM patients. This applied to patients who used insulin or oral antihyperglycaemic agents, and not to diet‐controlled DM patients. The same applied to hypertensive patients, who were at a higher risk of hypertension (0.54 vs. 0.29, *p* = 0.004). Dosing regimens are not detailed, but there is a reference to a guideline to manage SSNHL patients with 1 mg/kg daily of OST for 7–10 days [2].

In all, 10 papers reported no AEs [10, 14, 15, 16, 17, 18, 19, 20, 21, 22]. In these, the average daily dose of prednisolone was 42.5 mg, compared to 82.9 mg in papers reporting AEs (*p* = 0.33). Four papers reporting no AEs excluded diabetic patients, and one excluded any patients with contraindications to OST.

### 3.2 Intratympanic Steroid Therapy

20 ITS reports were identified.

A 25‐gauge spinal needle was the most common choice and was used in six papers. Postero‐inferior injections were the most common (*n* = 9), followed by antero‐inferior and antero‐superior (*n* = 3 each). One paper described injections in either the antero‐inferior or postero‐inferior quadrants, but rates were not detailed. Eight papers did not report needle size, and five did not report injection site.

The median number of doses was four (range 3–12), and the median time in which they were given was 14 days (range 3–28).

Dexamethasone was the most popular intratympanic drug. (*n* = 13) Methylprednisolone was second. (*n* = 6) Betamethasone and prednisolone were each used in one treatment arm.

Five papers performed secondary punctures to allow air in the middle ear to escape as the steroid solution was instilled. Two papers [18, 23] performed an antero‐inferior puncture, and two performed an antero‐superior puncture [19, 24] One paper performed a secondary myringotomy, but it was not detailed as to where this was [25].

Three papers documented that they warmed their steroid solutions [22, 26, 27]. Jia (2019) and Wang (2024) warmed them to body temperature, while Tong (2021) rolled the vial between hands.

The commonest AEs were temporary dizziness and otalgia. (Table 2) These were reported in nine and six papers respectively. The range of patients experiencing dizziness was 5.5%–27.1%. Dizziness was still present in studies which warmed their steroid solutions. 5.5% (*n* = 4 out of 73) of patients in Jia (2009) experienced vertigo, as did 20.0% (*n* = 6 out of 30) of patients in Tong (2021).

**TABLE 2:** Adverse events in intratympanic‐only study arms.

	Otalgia (%)	Dizziness (%)	Healing perf. (%)	Unspecified perf. (%)	Persistent perf. (%)	Severe otalgia (%)	Otitis media (%)
Labatut 2013	3/26 (11.5)	5/26 (19.2)			3/26 (11.5)		
Huang 2021	3/49 (6.1)	7.52 (13.5)					
Filipo 2013	3/106 (2.8)	22/106 (20.8)					
Anoop 2023	7/59 (11.9)	9/59 (15.3)		1/59 (1.7)			
Lyu 2020	3/7 (42.9)	1/7 (14.3)					
Rauch 2011	70/129 (54.3)*	35/129 (27.1)			5/129 (3.9)	2/131 (1.5)**	6/129 (4.7)
Swachia 2016	3/20 (15.0)						
Ermutlu 2017		4/19 (21.1)					
Tong 2021		6/30 (20.0)				1/31 (3.2)	
Kara 2010		5/29 (17.2)					
Jia 2009		4/73 (5.5)					
Tsai 2011			6/128 (4.7)				
Wang 2024			2/160 (1.3)				
Tsuda 2023					1/22 (4.7)		

* Patients reported both ‘ear pain’ and ‘injection pain’ as separate responses in this paper by Rauch (2011). We considered ‘injection pain’ as part of ‘ear pain’ and discounted the 35 separate cases of ‘injection pain’ due to potential overlap between the two causing artificially inflated numbers.

** Two patients withdrew consent due to severe otalgia and did not complete the treatment. However, we believe this should be reflected in our analysis, and therefore included them with the total patient number adjusted accordingly.

The range of patients experiencing otalgia was 2.8%–54.3%. Two papers reporting severe otalgia used methylprednisolone, while four of five papers reporting no otalgia used dexamethasone. Wang (2024) compared perceived pain levels between two injected concentrations of dexamethasone, methylprednisolone, and betamethasone. Post‐injection otalgia was significantly higher with betamethasone and more persistent with methylprednisolone. Local anaesthetic administration prior to injection did not correlate to reduced pain.

Perforations were divided into those which healed spontaneously and those which persisted beyond follow‐up. Several tympanoplasty methods are detailed, but there were patients who elected not to have further procedures. Healing perforations were noted in two papers in 1.3% and 4.7% of participants [23, 27] Persistent perforations are described in 3.9%–11.5% of studies, and all of these studies delivered 4 doses. Of the five studies which used secondary ventilation punctures, only Tsai (2010) reported a small number (4.7%) of healing perforations. We were unable to correlate the gauge of needle used, nor the number of injections, to the frequency of temporary and persistent perforations.

Seven studies report no AEs with ITS [4, 13, 18, 21, 24, 25, 28]. Most studies used dexamethasone except Fitzgerald (2007), who used methylprednisolone. Five studies used topical anaesthetic (lidocaine, phenol and one unspecified) and none warmed the steroid. Dispenza (2011) excluded any patients with a history of ear pathology and contraindication to steroids, and Hong (2009) excluded any DM patients. AE reporting methods were unreported in five papers. Han (2009) measured capillary blood glucose four times daily, and Dispenza (2011) captured AEs with an online patient questionnaire. The median number of injections for these papers was 3 (range: 3–8 doses) and the median period of administration was 14 days (range: 8–28 days), which is not significantly different from the wider cohort.

### 3.3 Intravenous Steroid Therapy

We identified 14 IVST study arms.

Studies preferred dexamethasone (*n* = 5) or prednisolone (*n* = 4). The median length of therapy (including oral tapers) was 10 days (range: 5–15 days). Ten studies included an oral taper and the average cumulative dose was 758.4 mg (range 266.7–2365 mg). The average daily dose was 84.6 mg (range 26.7–236.5 mg). Doses were given per weight in four papers and tapers were left undisclosed in two.

Hyperglycaemia was the most reported AE, and rates ranged from 11.9% to 48.0%. Lan (2018) examined a cohort of DM patients and likely monitored blood glucose levels closer than other studies. Uncontrolled hyperglycaemia occurred in 6.5% of one study, and one of these patients was subsequently removed from analysis. Insomnia was reported in 16.1%–41.3% of participants, and gastric disturbance in 2.2%–21.4%. (Table 3).

**TABLE 3:** Adverse events in intravenous‐only study arms.

	Hyperglycaemia (%)	Hypertension (%)	Gastric disturbance (%)	Insomnia (%)	Uncontrolled hyperglycaemia (%)	Severe infection (%)
Han 2009	4/30 (13.3)				2/31 (6.5)	
Song 2021 ‐ conventional				5/31 (16.1)		
Song 2021 ‐ high dose				4/21 (19.0)		
Kakehata 2006	4/21 (19.0)					
Tsuda 2023		2/46 (4.3)	1/46 (2.2)	19/46 (41.3)		
Plontke 2024	12/101 (11.9)					1/101 (0.9)
Sookdee 2022 ‐ Dex			3/14 (21.4)			
Lan 2018	24/50 (48.0)*					

* This study examined a cohort of diabetic patients with a higher propensity for steroid‐induced hyperglycemia and involved more careful monitoring.

Six papers reported no AEs. Among these, the average length of therapy was 10.5 days, and the average dose of prednisolone was 818.3 mg. One paper [29] disclosed participants' comorbidities, and no papers disclosed AE reporting methodology. Two papers [30, 31] excluded diabetic and hypertensive patients. Two papers did not report exclusion criteria. Four papers detailed therapies including a low salt diet (*n* = 3) and smoking cessation (*n* = 2).

### 3.4 Combination Therapies (Intratympanic Plus Systemic Therapies)

In all, 12 study arms used a combination of systemic steroid and ITS. This comprised six OST and ITS, and six IVST and ITS arms.

Studies most frequently used prednisolone or methylprednisolone (*n* = 3 each) as OST, dexamethasone (*n* = 3) for IVST, and dexamethasone for ITS (*n* = 10). Systemic therapy was given for 10.3 days on average in OST, and 10.8 days on average in IVST. Average daily doses were 53.0 mg and 51.9 mg respectively. The mean number of IT injections was 4.8 delivered over 11.2 days on average.

Vertigo and otalgia were the most reported AEs, with rates of 8.1%–28.3% and 3.8%–13.5% respectively. (Table 4) Perforations occurred transiently in 5.4% and persisted in 4.0% of patients. There was one case of otitis media (2.2%). One paper [32] reported on systemic AEs, and rates of hypertension and hyperglycaemia were in keeping with other OST or IVST reports. Appetite change and dyssomnia were markedly higher at 48.1% and 73.1% respectively. Tapers were estimated in 5 papers [33, 34, 35, 36, 37].

**TABLE 4:** Adverse events in intratympanic and systemic steroid study arms.

	Vertigo (%)	Otalgia (%)	Persistent perf. (%)	Transient perf. (%)	Otitis media (%)
Alexander 2015				2/37 (5.4)	
Battista 2005			1/25 (4.0)		
Gundogan 2013	3/37 (8.1)	5/37 (13.5)			
Huang 2021	8/52 (15.4)	2/52 (3.8)			
Koltsidopoulos 2013	13/46 (28.3)				1/46 (2.2)
Xie 2023	33/311 (10.6)	20/311 (6.4)			

In total, 5 papers noted no AEs [4, 29, 30, 37, 38]. Three papers append qualifiers—Arslan (2011) states no ‘important’ complications, Battaglia (2008) states no ‘long‐term’ complications and Fu (2011) states no ‘severe’ complications. Among these papers, the average daily dose of prednisolone was 54.5 mg, which is close to the average of 51.2 mg among papers reporting AEs.

### 3.5 Steroids in Pregnancy

We included papers related to pregnancy as this is an important demographic for clinicians to be aware of [24, 28, 39]. These papers used dexamethasone ITS and topical anaesthesia pre‐injection. All patients in one study received intravenous Dextran‐40 [28]. No complications were noted in two papers [24, 28]. 42.9% (*n* = 3) and 14.3% (*n* = 1) of seven patients enrolled in Lyu (2020) experienced post‐injection otalgia and vertigo respectively. No systematic AEs were reported, though it is difficult to draw conclusions due to the limited number of participants.

## 4 Discussion

This review has identified several risks to consider when starting steroid therapy to manage SSNHL. The incidence varies depending on the route of administration and can be divided into local and systemic AEs. Ranges are summarised in Table 5. While a proportion of studies reported no AEs, drawing conclusions is difficult due to variation in follow‐up, reporting measures, definitions of AEs, and additional therapies.

**TABLE 5:** Summary of adverse events grouped by route of administration.

	Side effect	PO (%)	IV (%)	IT (%)	Combination (%)
Systemic	Hyperglycemia	2.1–29.8	11.9–48.0		23.1
	Hypertension	4.2–37.9	4.3		13.5
	Gastric disturbance	4.2–27.9	2.2–21.4		48.1
	Mood or sleep disturbance	2.3–44.6	16.1–41.3		73.1
Local	Otalgia			6.1–54.3	3.8–13.5
	Dizziness			5.5–27.1	8.1–28.3
	Transient perf.			1.3–4.7	5.4
	Persistent perf.			3.9–11.5	4.0
	Infection			4.7	2.2

Long‐term steroids are used in the management of myriad inflammatory and auto‐immune conditions. In a survey of 2446 patients using long‐term glucocorticoids, Curtis et al. (2006) found that 90% of patients reported at least one AE, with 55% classifying it as ‘very bothersome’. These included weight gain (70%), cataracts (15%) and easy bruising (12%) and have a linear relationship with steroid dose and duration [9]. Sequelae such as bruising, osteoporosis, and avascular necrosis were not identified in our review and suggests that risk rises with long‐term usage or comorbidity. While the latter is a very rare AE, it carries significant impacts on a patient's life and requires operative intervention. Comorbid and immunosuppressed individuals are at greater risk, and should be counselled accordingly [40].

Clinicians should consider adrenal suppression caused by corticosteroid prescriptions. In prolonged courses, dose tapering allows for endogenous function to resume, but evidence to support an exact threshold is limited, especially in SNHL and ENT contexts. The literature suggests that steroid courses (of any dose) lasting under 2 weeks do not lead to long‐term suppression. Caution should be taken in patients who receive frequent short courses of steroids (e.g., for asthma) [41]. The Royal College of Physicians suggests courses of ≥ 40 mg of prednisolone for longer than 1 week are at risk of adrenal suppression, and patients should be given an NHS Steroid Emergency Card. This resource details the steroid therapy and contains guidance on management of adrenal crisis [42]. ENT UK guidelines recommend a tapering course of oral steroids, but further investigation is needed to correlate the need for a taper and clinical outcomes [1]. NICE guidelines suggest that proton‐pump inhibitors (PPI) need not be prescribed routinely but ‘considered for those at risk of gastrointestinal bleeding or dyspepsia’ for long‐term prescriptions [43]. A review by Narum et al. (2014) suggests that outpatient OST is not associated with an increased risk of gastrointestinal bleeding [44]. While these AEs may be avoided with ITS, delivery is dependent on local expertise and equipment. In these cases, patients should be carefully counselled on risks such as persistent tympanic membrane perforation to gain informed consent.

## 5 Conclusion and Recommendations

1. Closer ambulatory monitoring of diabetic and hypertensive patients. Blood pressure and glucose measurements can be monitored at home and discussed with general practitioners and local diabetic teams to titrate medications appropriately.
2. ITS as first‐line therapy requires further investigation in diabetic and hypertensive cohorts.
3. Gastroprotective measures should be considered in patients predisposed to gastrointestinal bleeding or dyspepsia.
4. Further well‐designed studies examining AEs and at‐risk cohorts are required to establish our clinical evidence base and improve counselling.
5. Develop patient information leaflets to include with urgent prescriptions which explain known side effects of steroid therapy.
6. Consider providing patients with the NHS steroid emergency card when prescribed a course of OST ≥ 40 mg daily for over 1 week.

The evidence base which clinicians can draw on to counsel their patients on the risk profile of short‐course steroids is limited. To effectively counsel our patients, further research is required.

### 5.1 Limitations

The greatest limitation to the conclusions made by this study is the considerable heterogeneity between papers. To allow for analysis, we estimated steroid tapers and an average weight of 70 kg where taper data were omitted, or doses were presented in mg per kg.

We assumed the length of follow‐up based on the date of the final audiology appointment unless specified.

Many papers excluded patients with diagnoses of diabetes or hypertension, or with unspecified contraindications to steroids. AE profiles may therefore be positively skewed. Some reports terminated steroid therapy following hearing recovery, but patients remained enrolled without completing the full course. This also potentially positively skews AE rates.

Different systolic blood pressure or blood glucose thresholds triggered AE recording. Immediately apparent events such as vertigo and otalgia are more easily captured compared to AEs such as hypertension or hyperglycaemia, which require additional equipment. In the ITS cohort, limited data made it difficult to correlate the gauge of spinal needle and perforation rates.

We converted all steroids to prednisolone‐equivalent doses. It is unclear whether the severity of AEs translates linearly. Some studies employed measures including dietary modification, hyperbaric oxygen, ginkgo biloba and Dextran; it is unclear whether these conferred any therapeutic effect.

## Author Contributions

M.A., P.K., M.F.E., T.R., T.M. and J.G.M. were all substantially involved in the following: – Substantially contributed to the conception and design of this review. – Drafting the work and revising it critically for important intellectual content. – Are in agreement to be accountable for all aspects of the work in ensuring that questions related to the accuracy or integrity of any part of the work are appropriately investigated and resolved.

## Ethics Statement

The authors have nothing to report.

## Conflicts of Interest

The authors declare no conflicts of interest.

## Peer Review

The peer review history for this article is available at https://www.webofscience.com/api/gateway/wos/peer‐review/10.1111/coa.14339.

## Supporting information


**Table S1.** Demographics of all study arms.

**Table S2.** Demographics of oral steroid‐only arms.

**Table S3.** Demographics of intratympanic steroid‐only arms.

**Table S4.** Demographics of intravenous steroid‐only arms.

**Table S5.** Demographics of combined steroid therapy arms.

**Table S6.** Demographics of steroid therapy in pregnancy‐only arms.

## Data Availability

Data sharing not applicable to this article as no datasets were generated or analysed during the current study.
