# Supplementary material for: Adverse Effects of Steroid Therapy in Sudden Sensorineural Hearing Loss: A Scoping Review
Source: Clin Otolaryngol. 2025 May 30;50(5):821–30. doi: 10.1111/coa.14339 (PMC12319462; doi:10.1111/coa.14339)
Supplement: Supplementary file 1 — Table S1. Demographics of all study arms. [file COA-50-821-s003.docx]

| **First author, year** | **PO arm** | **IT arm** | **IV arm*** | **PO and IT** | **IV and IT** | **Type of study** | **Prospective or retrospective?** | **SE data collection method** | **Length of follow up** | **Total number of participants** | **Co-morbidities** | **Mean age** |
| --- | --- | --- | --- | --- | --- | --- | --- | --- | --- | --- | --- | --- |
| Ahn 2008 |  |  |  |  |  | RCT | Prospective | Unreported | 3 months | 120 | Unreported | 47.3 |
| Alexander 2015 |  |  |  |  |  | Case series | Retrospective | Unreported | Unreported | 37 | Unreported | 60.3 |
| Anoop 2023 |  |  |  |  |  | Case series | Prospective | Unreported | 36 months | 59 | Unreported | 60.4 |
| Arslan 2011 |  |  |  |  |  | RCT | Prospective | Unreported | Unreported | 158 | Unreported | 48.1 |
| Battaglia 2008 |  |  |  |  |  | RCT | Prospective | Unreported | 3 months | 51 | Diabetes 3.9% | 56.9 |
| Battista 2005 |  |  |  |  |  | Case series | Prospective | Unreported | 6 months | 25 | Unreported | 53 |
| Choi 2014 |  |  |  |  |  | Non-randomised trial | Prospective | Medical chart review, insomnia, abdominal discomfort, HTN, hyperglycaemia necessitating medication initiation or modification documented alongside major complications e.g. myocardial infarction, GI bleeding, death | 3 months | 109 | Diabetes 23.9%  HTN 51.4% | 71.7 |
| Dispenza 2011 |  |  |  |  |  | RCT | Retrospective | Online Questionnaire | N/A | 908 | Unreported | 50 |
| Ermutlu 2017 |  |  |  |  |  | RCT | Prospective | The tympanic membrane was regularly checked until the perforation was healed. Case report form collected from each patient including side effects and complications. | 3 months | 35 | Unreported | 45.7 |
| Filipo 2013 |  |  |  |  |  | Case series | Prospective | Unreported | 1 year | 106 | Unreported | 50.7 |
| Fitzgerald 2007 |  |  |  |  |  | Case series | Retrospective | Unreported | Unreported | 21 | Unreported | 54.7 |
| Fu 2011 |  |  |  |  |  | Non-randomised trial | Prospective | Unreported | 1 month | 66 | Unreported | 38.7 |
| Fu 2019 |  |  |  |  |  | Case series | Prospective | Unreported | 2 months | 6 | Unreported | 30.2 |
| Gundogan 2013 |  |  |  |  |  | RCT | Prospective | Unreported | 4 weeks | 73 | Unreported | 52 |
| Halevy 2022 |  |  |  |  |  | Cohort study | Retrospective | AE defined as deviation from safe results (glucose, systolic BP, diastolic BP parameters given) which mandate initiation or modification of anti-hypertensive or anti-hyperglycaemic regimen, or halting systemic steroidal therapy and initiating intratympanic therapy. All ISSNHL patients are hospitalised with tight monitoring of BP TDS and glucose every morning before breakfast. | Unreported | 143 | Diabetes 20%  HTN 32% | 58 |
| Han 2009 |  |  |  |  |  | Non-randomised trial | Prospective | QDS blood glucose monitoring for those with uncontrolled hyperglycaemia | 8 weeks | 114 | Diabetes 100% | 58.2 |
| Hong 2009 |  |  |  |  |  | RCT | Prospective | Unreported | 3 months | 63 | Unreported | 56.6 |
| Huang 2021 |  |  |  |  |  | RCT | Prospective | Recorded in hospital and in outpatient clinics. | 3 months | 98 | Diabetes 24.5%  HTN 16.3% | 52.1 |
| Jia 2019 |  |  |  |  |  | Cohort study | Retrospective | Unreported | Unreported | 73 | Diabetes 100% | 54.7 |
| Kakehata 2006 |  | ** |  |  |  | Non-randomised trial | Prospective | Venous blood was used to measure free blood sugar before and after treatment. In addition, in the IV-Dex group, patients were treated with insulin according to FBS measured from capillary blood four times a day during the treatment. | Up to 60 months | 21 | Unreported | 60.4 |
| Kara 2010 |  |  |  |  |  | Non-randomised trial | Prospective | Unreported | 2 months | 60 | Unreported | 39.2 |
| Kim 2020 |  |  |  |  |  | Non-randomised trial | Prospective | Unreported | 2 months | 66 | Cardiovascular disease 9.1%  Diabetes 30.3%  HTN 37.9% | 50.4 |
| Koltsidopoulos 2013 |  |  |  |  |  | RCT | Prospective | Unreported | 3 months | 92 | Unreported | 55.7 |
| Koo 2016 |  |  |  |  |  | RCT | Prospective | Safety evaluated by interview and physical examination during the administration period + on days 3, 5, 14, and 28 of administration. In serious safety issues, subject unblinded, withdrawn from study and investigated. | 28 days | 24 | Unreported | 46.5 |
| Labatut 2013 |  |  |  |  |  | Case series | Prospective | Unreported | 3-6 months | 26 | Unreported | 53 |
| Lan 2018 |  |  |  |  |  | Cohort study | Retrospective | Co-morbidities, fasting blood sugar levels, DM medications, acute complications (HHS, DKA) noted. Clinical examinations performed in all patients. | 3 months | 50 | Diabetes 100% | 59.2 |
| Lee 2016 |  |  |  |  |  | Case series | Prospective | Unreported | 8 weeks | 229 | Diabetes 19.7% | 54.3 |
| Liu 2023 |  |  |  |  |  | Non-randomised trial | Prospective | Unreported | 2 months | 52 | Unreported | 44.6 |
| Lyu 2020 |  |  |  |  |  | Non-randomised trial | Prospective | Unreported | Unreported | 11 | Pregnancy 100% | 31.2 |
| Park 2011 |  |  |  |  |  | RCT | Prospective | Patient complaints were obtained at each follow up visit. Tympanic membrane photo-documented. Discomfort (otalgia, dizziness, tinnitus) recorded using VAS. >7 = serious complaint | 3 months | 88 | Diabetes 22.7% | 46.7 |
| Plontke 2024 |  |  |  |  |  | RCT | Prospective | 24-hour ambulatory blood pressure measurement, insulin and glucose measurements to assess for insulin resistance. Special safety interest events- hyperglycaemia during follow up visits, steroid-induced psychosis. | 180 days | 308 | Atrial fibrillation 3.6%  Coronary heart disease 7.5%  Diabetes 12.3%  HTN 45.5%  Previous MI 6.5%  Previous stroke 1.6% | 55.5 |
| Rauch 2011 |  |  |  |  |  | RCT | Prospective | Adverse events collected at follow up. Extensive review of systems questionnaire and visual analogue pain scale completed at each visit. In addition to checking vital signs and otological physical examination at each visit, safety monitoring laboratory studies included complete blood cell count, serum glucose measurement and urinalysis. Other safety testing was performed at the discretion of the treating physician. Adverse events and serious adverse events were assessed at all study visits. | 6 months | 250 | Unreported | 50.9 |
| Slattery 2005 |  |  |  |  |  | Cohort study | Retrospective | Unreported | Unreported | 75 | Unreported | 51.8 |
| Song 2021 |  |  |  |  |  | Non-randomised trial | Prospective | Unreported | 2 weeks | 52 | Diabetes 17.3%  HTN 32.7% | 52.3 |
| Sookdee 2022 |  |  |  |  |  | RCT | Prospective | Noted as a secondary outcome but no specifics. | 17 days | 28 | Unreported | 43.5 |
| Swachia 2016 |  |  |  |  |  | RCT | Prospective | Patients advised to report any adverse event during the study period. | 60 days | 42 | Diabetes 11.9% | 44.3 |
| Tong 2021 |  |  |  |  |  | RCT | Prospective | Unreported | 1 month | 90 | Unreported | 42.9 |
| Tsai 2011 |  |  |  |  |  | Case series | Retrospective | Chart review | 6-37 months (mean 11.4) | 128 | Diabetes: 85.2%  HTN 84.4% | 57 |
| Tsuda 2023 |  |  |  |  |  | Non-randomised trial | Prospective | "We assessed the side effects of each treatment in all the cases" but no specific methods | 8 months | 68 | Unreported | 62.2 |
| Wang 2024 |  |  |  |  |  | Cohort study | Retrospective | Data on pain, vertigo/dizziness, TM perforation collected. | >1 month | 160 | Unreported | 49.1 |
| Xie 2023 |  |  |  |  |  | RCT | Prospective | General ENT examination + otoendoscopy at each follow up visit | 6 months | 702 | Unreported | 43.8 |
| Xu 2019 |  |  |  |  |  | Cohort study | Retrospective | Unreported | 6 months | 16 | Pregnancy 100% | 27.7 |
| Yu 2019 |  |  |  |  |  | Cohort study | Retrospective | Unreported | 3 months | 129 | Diabetes 30.2%  HTN 31.0% | 55.5 |

Supplementary table 1: Demographics of all study arms.
