# Supplementary material for: Adverse Effects of Steroid Therapy in Sudden Sensorineural Hearing Loss: A Scoping Review
Source: Clin Otolaryngol. 2025 May 30;50(5):821–30. doi: 10.1111/coa.14339 (PMC12319462; doi:10.1111/coa.14339)
Supplement: Supplementary file 2 — Table S2. Demographics of oral steroid‐only arms. [file COA-50-821-s002.docx]

| **First author, year** | **No. of participants** | **Drug** | **Duration of therapy (days)** | **Cumulative dose in mg (assume 70kg)** | **Average daily dose (mg)** | **Pred equivalent (mg)** | **Length of follow up** | **Methods of AE reporting** | **Relevant exclusion criteria** | **Additional therapies** |
| --- | --- | --- | --- | --- | --- | --- | --- | --- | --- | --- |
| Liu 2023 | 52 | PRED | 10 | 600 | 60 | 60.0 | 2 months (OP) | Unreported | Diabetes, severe hypertension | None |
| *Swachia 2016* | *22* | *PRED* | *14* | *690* | *49.3* | *49.3* | *60 days (OP)* | *Patients encouraged to report* | *Pregnant women* | *None* |
| *Koo 2016* | *24* | *MPD* | *12* | *440* | *36.7* | *45.8* | *28 days (OP)* | *History and examination* | *Severe renal/hepatic/respiratory disease or severe heart failure,pregnancy, breast-feeding,* | *None* |
| Xie 2023 | 391 | PRED | 5 | 300 | 60.0 | 60.0 | 6 months (IP+OP) | Patients examined at follow-up appointments | Diabetes, gastrointestinal ulcers, mental disorders, epilepsy | Gingko biloba extract, Vitamin B1, and mecobalamin tablets for 14 days, mannitol for ascending type-hearing loss, batroxobin for flat-type and profound hearing loss. |
| Gundogan 2013 | 36 | MPD | 14 | 480 | 34.3 | 42.9 | 4 weeks (IP+OP) | Unreported | None relevant | Low-salt diet, PPI |
| *Han 2009* | *48* | *PRED* | *10* | *430* | *43.0* | *43.0* | *8 weeks (IP +OP)* | *Blood glucose monitoring for high risk patients* | *None relevant* | *Took rest, quit smoking, low-salt diet, trimetazidine 50mg TDS, gingko bilobba extract 80mg BD* |
| *Rauch 2011* | *121* | *PRED* | *19* | *990* | *52.1* | *52.1* | *6 months (OP)* | *Questionnaire, examinations, bloods and urine analysis* | *Previous ear surgery, insulin-dependent diabetes mellitus, rheumatic disease, active atherosclerotic vascular disease, severe osteoporosis* | *None* |
| Yu 2019 | 129 | PRED | 11 | 468 | 42.5 | 42.5 | 3 months (OP) | Unreported | None relevant | None |
| Slattery 2005 | 75 | PRED | 14 | 645 | 46.1 | 46.1 | Unreported (OP) | Unreported | None relevant | None |
| Dispenza 2011 | 21 | PRED | 14 | 510 | 36.4 | 36.4 | 6 months (OP) | Online questionnaire | Any contraindication to systemic steroid administration | None |
| *Choi 2014* | *66* | *MPD* | *14* | *432* | *30.9* | *38.6* | *3 months (IP +OP)* | *Medical chart review* | *Uncontrolled hypertension or diabetes mellitus* | *Continuous infusion of 10 µg/d alprostadil over 7 days or daily infusions of 88 mg of zinc sulfate hydrate were provided.* |
| *Choi 2014* | *43* | *MPD (low dose)* | *8* | *144* | *18.0* | *22.5* |  |  |  |  |
| Ermutlu 2017 | 16 | PRED | 18 | 630 | 35.0 | 35.0 | 3 months (OP) | Patients examined at follow-up appointments, case report form detailed side effects and complications. | None relevant | Low molecular dextran for 5-10 days; acetazolamide for 1 month; betahistine; trimetazidine for 3 months |
| *Plontke 2024* | *105* | *DEX* | *5* | *200* | *40.0* | *400.0* | *180 days (OP)* | *24-hour ambulatory blood pressure measurement, insulin and glucose measurements to assess for insulin resistance. Special safety interest events* | *None relevant* | *None* |
|  | *102* | *PRED* | *10* | *450* | *45.0* | *45.0* |  |  |  |  |
| *Kara 2010* | *31* | *MPD* | *10* | *400* | *40.0* | *50.0* | *2 months (IP+OP)* | *Unreported* | *None relevant* | *Antiviral agents, low molecular weight dextran, anti-aggregants and vitamins. Low salt diet, antacid treatment.* |
| Ahn 2008 | 60 | MPD | 14 | 544 | 38.9 | 48.6 | 3 months (IP +OP) | Unreported | None relevant | Low salt diet, prostaglandin E1 |
| Hong 2009 | 31 | PRED | 8 | 360 | 45.0 | 45.0 | 3 months (OP) | Unreported | Diabetes | Peripheral vasodilator, gingko biloba extract |
| Tong 2021 | 30 | MPD | 10 | 280 | 28.0 | 35.0 | 1 month (OP) | Unreported | None relevant | Low salt diet, instructed to stop drinking alcohol and smoking |
| *Halevy 2022* | *143* | *Specifics unreported.* |  |  |  |  | *Unreported (IP)* | *BP and blood glucose monitoring* | *Unreported* | *None* |

Supplementary table 2: Demographics of oral-steroid only arms.
