# Supplementary material for: Adverse Effects of Steroid Therapy in Sudden Sensorineural Hearing Loss: A Scoping Review
Source: Clin Otolaryngol. 2025 May 30;50(5):821–30. doi: 10.1111/coa.14339 (PMC12319462; doi:10.1111/coa.14339)
Supplement: Supplementary file 3 — Table S3. Demographics of intratympanic steroid‐only arms. [file COA-50-821-s004.docx]

| **First author, year** | **No. of participants** | **Drug** | **Dose (mg)** | **Total doses** | **Duration of therapy (days)** | **Needle size** | **Ventilation site** | **Anesthetic** | **Solution warmed** | **Length of follow up** | **Methods of AE reporting** | **Relevant exclusion criteria** | **Additional therapies** |
| --- | --- | --- | --- | --- | --- | --- | --- | --- | --- | --- | --- | --- | --- |
| Battaglia 2008 | 17 | DEX | 7.2 | 3 | 21 | Unknown | N/A | Unknown | Unknown | 3 months (OP) | Unreported | None relevant | None |
| Swachia 2016 | 20 | MPD | 40 | 4 | 14 | 26 | N/A | Topical 4% xylocaine on cotton pledget | Unknown | 60 days (OP) | Patients encouraged to report | Pregnant women | None |
| *Tsai 2011* | *128* | *DEX* | *2.5* | *4* | *14* | *25* | *AI* | *Unknown* | *Unknown* | *6-37 months (OP)* | *Chart review* | *None relevant* | *None* |
| Han 2009 | 34 | DEX | 2.5 | 4 | 14 | 25 | N/A | Topical 10% lidocaine spray | Unknown | 8 weeks (IP +OP) | Blood glucose monitoring for high risk patients | None relevant | Took rest, quit smoking, low-salt diet, trimetazidine 50mg TDS, gingko bilobba extract 80mg BD |
| *Jia 2009* | *73* | *MPD* | *40* | *5* | *10* | *Unknown* | *N/A* | *Topical 1% tetracaine* | *Yes* | *Unreported* | *Unreported* | *Haemorrhagic disorders, pregnancy, peptic ulcer, other severe systemic disease* | *None* |
| Tsuda 2023 | 22 | DEX | 3.3 | 4 | 11 | Unknown | N/A | Iontophoresis on first visit, then diethylaminoethyl-p-butylaminobenzoate hydrochloride on subsequent visits | Unknown | 8 months (OP) | "We assessed the side effects of each treatment in all the cases" but no specific methods | None relevant | PPI, vitamin B12 and oral adenosine 5'-triphosphate disodium hydrate. |
| *Labatut 2013* | *26* | *MPD* | *40* | *4* | *14* | *27* | *N/A* | *Injected 1% lidocaine 1:100,000 adr to vascular strip* | *Unknown* | *3-6months (OP)* | *Unreported* | *Previous otologic surgery* | *None* |
| Dispenza 2011 | 25 | DEX | 2 | 4 | 28 | Unknown | AI | Unknown | Unknown | 6 months (OP) | Online questionnaire | Any contraindication to systemic steroid administration | None |
| *Huang 2021* | *49* | *DEX* | *1.5* | *12* | *24* | *Unknown* | *N/A* | *Topical 1.5% tetracaine* | *Unknown* | *3 months (IP+OP)* | *Chart review* | *Unmanaged hypertension, diabetes, epilepsy or psychosis, active ulcer, meniere's disease, large vestibular aqueduct syndrome* | *None* |
| *Filipo 2013* | *106* | *PRED* |  | *3* | *3* | *25* | *N/A* | *Topical 10% lidocaine on cotton* | *Unknown* | *1 year (OP)* | *Unreported* | *Previous otological surgery, presence of superior canal fistula* | *None* |
| *Ermutlu 2017* | *19* | *DEX* | *2.4* | *3* | *6* | *27* | *AS* | *Topical 10% lidocaine on cotton* | *Unknown* | *3 months (OP)* | *Patients examined at follow-up appointments, case report form detailed side effects and complications.* | *None relevant* | *Low molecular dextran for 5-10 days; acetazolamide for 1 month; betahistine; trimetazidine for 3 months* |
| *Kara 2010* | *29* | *DEX* | *2.4* | *5* | *5* | *25* | *N/A* | *Unknown* | *Unknown* | *2 months (IP+OP)* | *Unreported* | *Previous otologic surgery* | *Antiviral agents, low molecular weight dextran, anti-aggregants and vitamins. Low salt diet, antacid treatment.* |
| *Wang 2024* | *40* | *DEX, DEX, MPD, BET* | *2.5, 5, 20, 2* | *6* | *14* | *25* | *N/A* | *Topical 2.4% lidocaine spray* | *Yes* | *> 1 months (OP)* | *Chart review and examination* | *History of otological surgery, acute/recurrent vertigo* | *None* |
| *Anoop 2023* | *59* | *DEX* | *4* | *8* | *28* | *Unknown* | *N/A* | *Unknown* | *Unknown* | *36 months (OP)* | *Unreported* | *Pregnancy, middle ear tumours* | *Pentoxifylline 400mg TDS, xantinol nicotinate 500mg BD given for 3 weeks* |
| Hong 2009 | 32 | DEX | 2 | 8 | 8 | 26 | N/A | Topical 10% xylocaine | Unknown | 3 months (OP) | Unreported | Diabetes | Peripheral vasodilator, gingko biloba extract |
| Fu 2019 | 6 | DEX | 4 | 3 | 7 | 25 | AS | Lidocaine (unsure if topical or injected)Topical lidocaine | Unknown | 2 months (OP) | Unreported | None relevant | None |
| *Tong 2021* | *30* | *MPD* | *24* | *5* | *10* | *22* | *N/A* | *Topical lidocaine 10% on cotton* | *Yes* | *1 month (OP)* | *Unreported* | *None relevant* | *Low salt diet, instructed to stop drinking alcohol and smoking* |
| Fitzgerald 2007 | 21 | MPD | 25 | 3 | 21 | 27 | Myringotomy made; unclear where | Topical 15% phenol | Unknown | Unreported (OP) | Unreported | Previous Otologic surgery | None |
| *Lyu 2020* | *7* | *DEX* | *2.5* | *4* | *8* | *27* | *N/A* | *Topical 2% lidocaine* | *Unknown* | *Unreported (OP)* | *Unreported* | *Otosclerosis, meniere's disease, large vestibular aqueduct syndrome, previous otologic surgery* | *None* |
| Xu 2019 | 16 | DEX | 2.5 | 3 | 6 | Unknown | N/A | Topical anesthesia (unspecified) | Unknown | 6 months (OP) | Unreported | Neuropsychiatric disease, acoustic trauma | 500mL of 10% Dextran-40 intravenously for 10 days |
| Rauch 2011 | 129 | MPD | 40 | 4 | 14 | Unknown | N/A | Topical phenol | Unknown | 6 months (OP) | Questionnaire, examinations, bloods and urine analysis | Previous ear surgery, insulin-dependent diabetes mellitus, rheumatic disease, active atherosclerotic vascular disease, severe osteoporosis | None |

Supplementary table 3: Demographics of intratympanic-steroid only arms.
