# Supplementary material for: Adverse Effects of Steroid Therapy in Sudden Sensorineural Hearing Loss: A Scoping Review
Source: Clin Otolaryngol. 2025 May 30;50(5):821–30. doi: 10.1111/coa.14339 (PMC12319462; doi:10.1111/coa.14339)
Supplement: Supplementary file 4 — Table S4. Demographics of intravenous steroid‐only arms. [file COA-50-821-s001.docx]

| **First author, year** | **No. of participants** | **IV Drug** | **Duration of IV therapy (days)** | **Oral step down** | **Duration of Oral therapy (days)** | **Cumulative dose in mg (assume 70kg)** | **Length of follow up** | **Methods of AE reporting** | **Relevant exclusion criteria** | **Additional therapies** |
| --- | --- | --- | --- | --- | --- | --- | --- | --- | --- | --- |
| Arslan 2011 | 73 | MPD | 1 | PRED | 9 | 525 | Unreported | Unreported | Contraindications to systemic steroids such as diabetes, hypertension and cardiovascular disease. | Low salt diet, dextran 4000 |
| *Song 2021* | *31* | *DEX* | *5* | *PRED* | *10* | *463.8* | *2 weeks (IP+OP)* | *Unreported* | *None relevant* | *Insulin and oral anti-diabetic drugs prescribed for diabetics with advice from endocrinology team. Kallidinogenase 150 IU/day + mecobalamin 1500 µg/day. When needed - Zolpidem tartrate 6.25mg OD for insomnia; ursodeoxycholic acid post-intervention for liver markers.* |
|  | *21* | *DEX* | *7* | *PRED* | *14* | *892.5* |  |  |  |  |
| Koltsidopoulos 2013 | 46 | PRED | 9 | PRED | 15 | 540 | 3 months (IP +OP) | Unreported | Unreported | Low salt diet, advised to stop smoking |
| Lee 2016 | 130 | PRED | 5 | PRED | 10 | 480 | 8 weeks (IP+OP) | Unreported | None relevant | None |
| *Han 2009* | *32* | *PRED* | *7* | *PRED* | *11* | *520* | *8 weeks (IP +OP)* | *Bloods glucose monitoring for high risk patients* | *None relevant* | *Took rest, quit smoking, low-salt diet, trimetazidine 50mg TDS, gingko bilobba extract 80mg BD* |
| *Kakehata 2006* | *21* | *DEX* | *10* | *N/A* | *10* | *266.7* | *Up to 60 months (OP)* | *Bloods glucose monitoring* | *None relevant* | *None* |
| *Tsuda 2023* | *46* | *HYD* | *7* | *N/A* | *7* | *1100* | *8 months (OP)* | *"We assessed the side effects of each treatment in all the cases" but no specific methods* | *None relevant* | *PPI, vitamin B12 and oral adenosine 5'-triphosphate disodium hydrate.* |
| *Plontke 2024* | *101* | *PRED* | *5* | *PRED* | *5* | *1250* | *180 days (OP)* | *24 hour ambulatory blood pressure and blood glucose and insulin measurement, special interest events* | *None relevant* | *None* |
| Fu 2011 | 44 | DEX | 9 | N/A | 9 | 600 | Unreported | Unreported | Unreported | Hyperbaric oxygen therapy, prostaglandin |
| Sookdee 2022 | 14 | MPD | 3 | PRED | 10 | 2365 | 17 days (OP) | Unreported | Diabetes mellitus, uncontrolled hypertension, gastrointestinal bleeding | None |
|  | *14* | *DEX* | *3* | *PRED* | *10* | *730* |  |  |  |  |
| *Lan 2018* | *50* | *HYD* | *3* | *PRED* | *14* | *485* | *Clinical examinations and blood glucose levels* | *Unreported* | *None relevant* | *None* |
| Tong 2021 | 30 | MPD | 10 | N/A | 10 | 400 | 1 month (OP) | Unreported | None relevant | Low salt diet, advised to stop smoking and drinking alcohol |

Supplementary table 4: Demographics of intravenous-steroid only arms.
