# Supplementary material for: Adverse Effects of Steroid Therapy in Sudden Sensorineural Hearing Loss: A Scoping Review
Source: Clin Otolaryngol. 2025 May 30;50(5):821–30. doi: 10.1111/coa.14339 (PMC12319462; doi:10.1111/coa.14339)
Supplement: Supplementary file 5 — Table S5. Demographics of combined steroid therapy arms. [file COA-50-821-s005.docx]

| **First author, year** | **No. of participants** | **Systemic drug** | **Duration of systemic therapy (days)** | **Cumulative dose in mg (assume 70kg)** | **IT Drug** | **IT Dose mg** | **Total doses** | **Time period (days)** | **Needle size** | **Ventilation site** | **Anesthetic** | **Solution warmed** | **Length of follow up** | **Methods of AE reporting** | **Relevant exclusion criteria** | **Additional therapies** |
| --- | --- | --- | --- | --- | --- | --- | --- | --- | --- | --- | --- | --- | --- | --- | --- | --- |
| Battaglia 2008 | 16 | PRED | 14 days | 660mg (for group B and C) | DEX | 7.2 | 3 | 15 | unreported | Unreported | None | Unreported | 3 months (OP) | Unreported | None relevant | None |
| *Battista 2005* | *25* | *MPD* | *11* | *440 (estimate)* | *DEX* | *7.2* | *4* | *14* | *27* | *Unreported* | *Phenol* | *Unreported* | *6 months (OP)* | *Unreported* | *None relevant* | *None* |
| *Xie 2023* | *391* | *PRED* | *if successful: 5 days, if unsuccessful: 3 days* | *successful: 300mg, unsuccessful: 180mg.* | *DEX* | *5* | *4* | *7* | *unreported* | *unreported* | *1% Tetracaine* | *Unreported* | *6 months (IP+OP)* | *Patients examined at follow-up appointments* | *Diabetes, gastrointestinal ulcers, mental disorders, epilepsy* | *Gingko biloba extract, Vitamin B1, and mecobalamin tablets for 14 days, mannitol for ascending type-hearing loss, batroxobin for flat-type and profound hearing loss.* |
| *Gundogan 2013* | *37* | *MPD* | *14* | *900* | *MPD* | *25* | *4* | *14* | *unreported* | *unreported* | *Lidocaine 10% spray* | *Unreported* | *4 weeks (IP+OP)* | *Unreported* | *None relevant* | *Low-salt diet, PPI* |
| *Kim 2019* | *66* | *MPD* | *11* | *600 (estimate)* | *DEX* | *unable to calculate* | *5* | *9* | *unreported* | *unreported* | *unreported* | *unreported* | *2 months (OP)* | *Unreported* | *Severe diabetes mellitus, active peptic ulcer, severe bleeding diathesis or previous Intracranial haemorrhage* | *None* |
| *Alexander 2015* | *37* | *PRED* | *14* | *575 (estimate)* | *DEX* | *13.5 or 18* | *4* | *14* | *27* | *unreported* | *with phenol or without* | *unreported* | *Unreported (OP)* | *Unreported* | *Menieres or autoimmune hearing loss* | *None* |
| Arslan 2011 | 85 | MPD (IV 1 day) PRED (Oral 8 days) | 9 | 525 | MPD | 62.5 | 5 | 10 | 22 | Unreported | None | Unreported | Unreported | Unreported | Contraindications to systemic steroids such as diabetes, hypertension and cardiovascular disease. | Low salt diet, dextran 4000 |
| *Koltsidopoulos 2013* | *37* | *PRED (IV 9 days, PO 6 days)* | *15* | *IV : 450, oral estimate: 90 ; total estimate: 540* | *DEX* | *2* | *3* | *5 (1,3 and 5th day of hospitalisation)* | *25* | *unreported* | *EMLA cream* | *yes* | *3 months (IP +OP)* | *Unreported* | *Unreported* | *Low salt diet, advised to stop smoking* |
| Park 2011 | 44 | IV DEX 7 days, PO pred taper for 3 days | 10 | IV known: 433.5, oral estimate: 90; total estimate 523.5 | DEX | 1.75 | 6 | 14 | 25 | unreported | 10% lidocaine spray | unreported | 3 months (IP+OP) | Tympanic membrane photo-documented. Discomfort (otalgia, dizziness, tinnitius) recorded using VAS. >7 = serious complaint | Known vertigo, diabetes, tumours | None |
| *Huang 2021* | *49* | *DEX* | *12* | *600* | *DEX* | *1.5* | *group a: 12, group b: 6* | *group a: 24 ; group b: 12* | *N/A* | *unreported* | *1.5% tetracaine* | *unreported* | *3 months (IP+OP)* | *Chart review* | *Unmanaged hypertension, diabetes, epilepsy or psychosis, active ulcer, meniere's disease, large vestibular aqueduct syndrome* | *None* |
| Fu 2011 | 22 | DEX | 9 | 600 | DEX | 4 | 5 | 9 | unreported | unreported | unreported | unreported | Unreported | Unreported | Unreported | Hyperbaric oxygen therapy, prostaglandin |
| Lee 2015 | 99 | PRED | 10 | 480 | DEX | 1.75 | 5 | 5 | 25 | AS | Lidocaine 10% spray | unreported | 8 weeks (IP+OP) | Unreported | Previous otologic surgery, retrocochlear lesions | None |

Supplementary table 5: Demographics of combined steroid therapy arms.
