# Supplementary material for: Adverse Effects of Steroid Therapy in Sudden Sensorineural Hearing Loss: A Scoping Review
Source: Clin Otolaryngol. 2025 May 30;50(5):821–30. doi: 10.1111/coa.14339 (PMC12319462; doi:10.1111/coa.14339)
Supplement: Supplementary file 6 — Table S6. Demographics of steroid therapy in pregnancy‐only arms. [file COA-50-821-s006.docx]

| **First author, year** | **No. of participants** | **Mean age** | **Additional therapies** | **Anaesthetic** | **Injection site** | **Complications** |
| --- | --- | --- | --- | --- | --- | --- |
| Lyu 2020 | 7 | 31.2 | None | Topical 2% lidocaine | AI or PI | Otalgia 42.9%  Vertigo 14.3% |
| Fu 2019 | 6 | 30.2 | None | Topical lidocaine (unspecified) | PI | No |
| Xu 2019 | 30 | 27.7 | 500mL 10% dextran infusion for 10 days | Topical anaesthesia (unspecified) | PI | No |

Supplementary table 6: Demographics of steroid therapy in pregnancy-only arms.
